# Microbial Distortion? Impacts of Delayed Preservation on Microbiome Diversity and Composition in a Marine Invertebrate

**DOI:** 10.1002/mbo3.70019

**Published:** 2025-05-15

**Authors:** Brenna Hutchings, Susanna López‐Legentil, Lauren Stefaniak, Marie Nydam, Patrick M. Erwin

**Affiliations:** ^1^ Department of Biology & Marine Biology, Center for Marine Science University of North Carolina Wilmington Wilmington North Carolina USA; ^2^ Department of Marine Science Coastal Carolina University Conway South Carolina USA; ^3^ Life Sciences Concentration Soka University of America Aliso Viejo California USA

**Keywords:** ascidian, bacteria, methodology, sea‐squirt, symbiosis

## Abstract

Field collections of marine invertebrates are often accompanied by delays in preservation, which may impact microbiome composition. Here, we tested the effects of delayed preservation and relaxation methods on microbiome diversity and composition in the colonial ascidian *Trididemnum solidum* using 16S rRNA amplicon sequencing. Replicate samples collected from Belizean reefs were either (1) immediately preserved in ethanol (“control”), (2) held in ambient seawater for 3 h before preservation (“SW”), or (3) held in ambient seawater with menthol (a common pre‐preservation relaxation technique for ascidian identification) for 3 h before preservation (“SW + M”). All *T. solidum* microbiomes were different from ambient seawater bacterioplankton and dominated by the same microbial taxa, including the genera *Thalassobaculum*, *Tistrella*, and *Synechocystis*. However, the 3‐h delay in sample preservation (SW) significantly reduced microbiome richness compared to controls (*p* = 0.028), while menthol treatment (SW + M) mitigated this diversity loss (*p* = 0.208). Microbial composition at the community level did not differ significantly for either delayed preservation method compared to controls (SW *p* = 0.054, SW + M *p* = 0.052). Taxon‐level shifts were rare but did occur, most notably a bloom of the facultatively anaerobic gammaproteobacterium *Catenococcus* that was 37x (SW) and 197x (SW + M) more abundant in delayed preservations. After a 3‐h preservation delay (SW), only 122 microbial taxa (1.85% of total) exhibited significantly differential abundances with controls, with menthol treatment (SW + M) reducing taxon‐level shifts to 65 taxa (0.98%). Our results showed that brief delays in preservation did not significantly alter community‐level microbiome composition and dominant taxa, with menthol exposure counteracting minor microbiome shifts associated with preservation delays.

## Graphical abstract

Delayed preservation of marine invertebrates during field sampling may impact microbiome characterization. Here, we show that a 3‐h preservation delay decreased microbiome richness in the ascidian *Trididemnum solidum*; however, menthol addition mitigated diversity loss and overall microbiome composition was not significantly altered, as dominant taxa remained intact. Thus, we report minimal impacts of short‐term preservation delay on ascidian microbiomes and show that the common practice of menthol treatment before preservation can counteract minor microbiome shifts associated with processing delays.

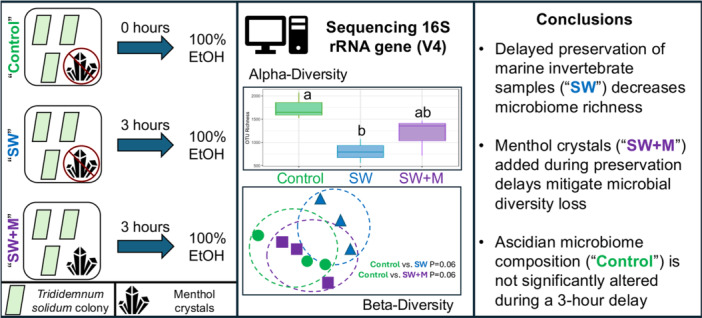

## 1 Introduction

When marine invertebrates are collected from their natural habitats, there may be a delay before the specimens can be preserved, often due to the collection sites being miles from the laboratory space. While a delay of a few hours may not affect invertebrate identification and processing, the microbial communities living in association with the invertebrate host may be altered. Some microbial taxa may exhibit rapid growth rates, while others may disappear due to reduced water flow and changes in oxygen levels, skewing microbial data and altering the abundances of culturable and nonculturable cells (Ferguson et al. 1984). Even when efforts are made to maintain conditions as close as possible to those in situ, after ~30 h the microbial communities within may display changes in dominant taxa and gene expression (Stewart et al. 2012). Thus, when and how marine invertebrate hosts will be preserved needs to be considered when the microbial communities within host tissues will be characterized.

The efficacy of host tissue preservation methods for later microbial analyses using DNA and RNA extractions has been assessed for various aquatic invertebrates. In marine sponges, for example, storage in RNAlater or liquid nitrogen were shown to be equally effective (Simister et al. 2011). In sea anemone hosts, both fresh tissue and tissue immediately frozen with liquid nitrogen were suitable for microbial community characterization (Rocha et al. 2014). Preservation of crayfish and aquatic insect larvae in ethanol was as efficient as freezing at −20°C for conserving gut microbial communities (Vaughn and Jackson 2022). While it appears that standard host preservation methods also preserve microbial taxa, delays before this preservation can occur may still impact microbial composition.

In ascidians (Class Ascidiacea, Phylum Chordata) or sea‐squirts, delays in preservation following collection are a common practice. Ascidians are benthic, sessile organisms, and when collected they rapidly contract their muscles, making species identification based on morphological characters impossible in most cases. To relax the ascidian before preservation, menthol crystals or liquid drops are commonly added to a holding tank or zip‐top collection bag of seawater for at least 2 h (Monniot et al. 1991; Stefaniak and Heupel 2016). Menthol in an aqueous state has an antibacterial effect due to its interaction with plasma membrane lipids, leading to the leakage of intracellular materials (Trombetta et al. 2005). A weak antifungal activity was also reported (İşcan et al. 2002). To our knowledge, no research has assessed the effect of menthol crystals on the microbiome of ascidians, despite menthol's documented history as an antimicrobial agent.

The microsymbionts in the tunic of the colonial ascidian *Trididemnum solidum* are well‐documented due to the discovery and isolation of bioactive marine natural products in the species. These compounds, called “didemnins,” are produced by the Pseudomonadota (previously Proteobacteria) *Tistrella mobilis* (Tsukimoto et al. 2011; Xu et al. 2012) and *T. bauzanensis* (Xu et al. 2012). Members of the *Trididemnum* genus also host the symbiotic cyanobacteria *Synechocystis* (Olson 1986) and *Prochloron* (Hirose et al. 2006; López‐Legentil et al. 2011). The symbiotic relationship between *Trididemnum* and cyanobacteria is obligate, as shading experiments have indicated that the survival *T. solidum* is dependent on the presence of *Synechocystis* (Olson 1986) and larvae of *T. strigosum* (Kott 1980) and *T. miniatum* (Hirose and Hirose 2007) host *Prochloron* cells inherited from the mother colony. While the genetic identity of *T. solidum* photosymbionts is well established (Stackebrandt et al. 1982; Münchhoff et al. 2007; López‐Legentil et al. 2011), no study to date has characterized the whole microbiome present in the animal or how this microbiome may be affected after a 3‐h preservation delay or menthol addition.

## 2 Materials and Methods

### 2.1 Ascidian and Seawater Collection

**Figure 1:**
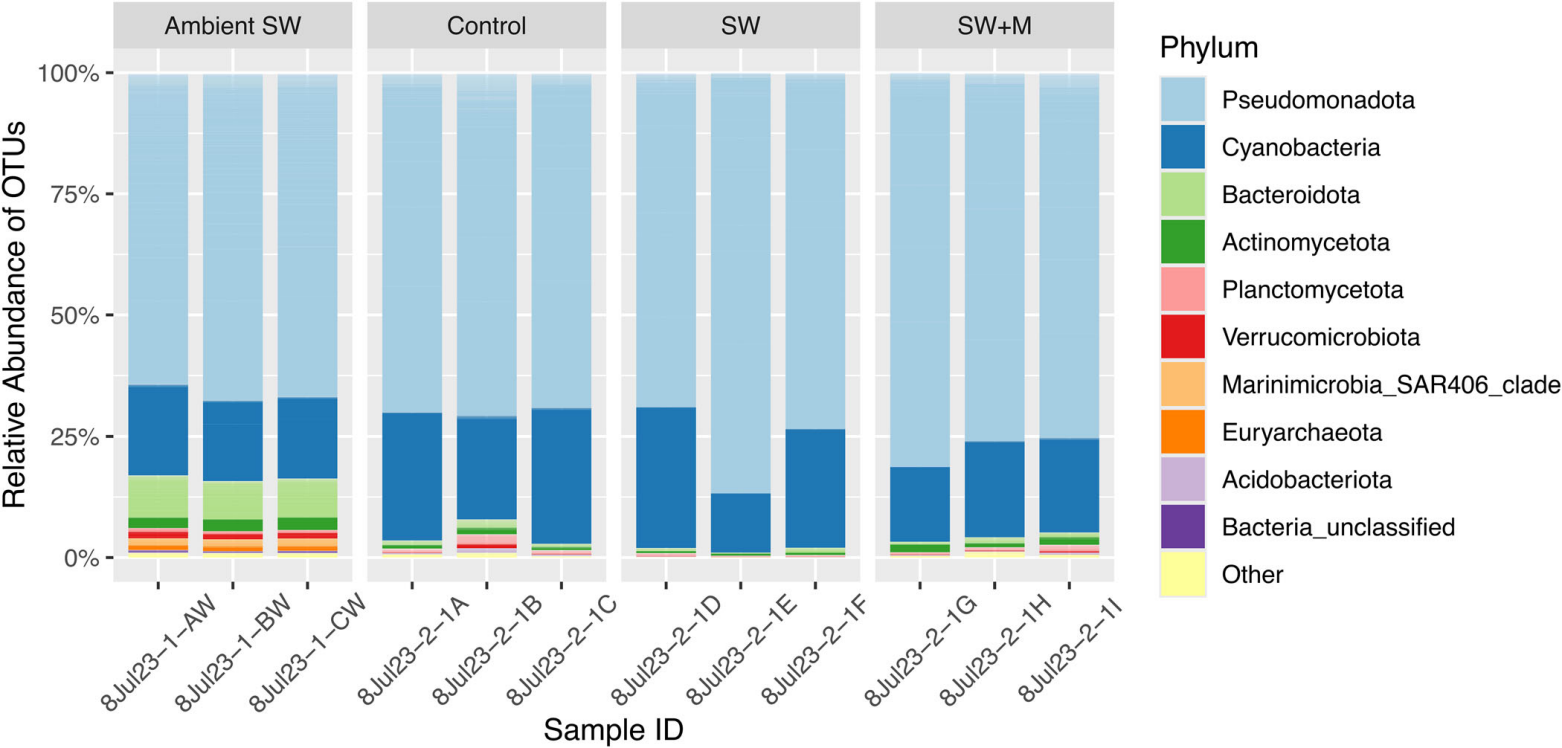
Phylum‐level composition of microbial communities in *T. solidum* (Control, SW, SW + M) and ambient seawater (Ambient SW), showing the relative abundance of the top nine most abundant taxa. The “Other” category represents all other phyla. Ascidian samples are grouped by preservation treatment: immediate (Control), 3‐h delay in seawater only (SW), and 3‐h delay in seawater and menthol (SW + M).

A single colony of *Trididemnum solidum* (Supporting Information S1: Figure S1A) was collected from a reef in Tobacco Caye, Belize (GPS: 16.897316, −88.058959) on July 8, 2023. The species genetic barcode was obtained following procedures described in Morrison et al. (2024) for the 18S rRNA gene (GenBank acc. num. PQ062521, PQ062522). The colony was transferred into a zip‐top collection bag of ambient seawater for travel back to Carrie Bow Cay Field Station (ca. 5 miles away). Once at Carrie Bow Cay, the colony was cut into nine pieces of approximately the same size using a sterile razor blade. Three of these pieces (Supporting Information S1: Figure S1B) were immediately fixed in 95% ethanol and will be referred to as the “control” specimens throughout the manuscript. Three pieces were kept in a cup of ambient seawater at room temperature for 3 h (Supporting Information S1: Figure S1C) and then immediately placed in 95% ethanol (“SW” treatment). The final three samples were kept in a cup of ambient seawater with menthol crystals at room temperature for 3 h (Figure 1C), then placed in 95% ethanol (“SW + M” treatment). Ethanol was changed daily for the next 3 days to prevent water content of tissue from diluting the preservative, and then samples were placed in 100% ethanol and stored at −20°C upon return to the USA, as is standard for long‐term storage of ascidian tissues. Three seawater replicates were also collected at the time of ascidian sampling (July 8, 2023) to characterize the bacterioplankton. For each replicate, 500 mL of seawater was filtered through a 0.2 μm filter with a Nalgene vacuum filtration system. Filters were stored in RNAlater and kept at −20°C for long‐term storage upon return to the USA. Preservation of prokaryotic cells in either ethanol or RNAlater in cold storage are equally effective for later molecular analyses (Bachoon et al. 2001).

### 2.2 Amplicon Sequencing and Microbiome Composition

A small piece (~1 mm^2^) of each *T. solidum* sample, encompassing both tunic and zooids, and approximately a third of each ambient seawater filter were dissected for DNA extraction using the DNeasy Blood and Tissue Kit (QIAGEN). To ensure the quality of DNA extractions, the V4 region of the 16S rRNA gene was amplified via polymerase chain reaction (PCR) using 515f and 806r primers (Caporaso et al. 2011). Each PCR reaction consisted of 0.5 µL of the forward and reverse primers, 11 µL of PCR water, 12.5 µL of MyTaq HS Red Mix, and 0.5 µL of DNA. The PCR process was conducted on an Eppendorf Mastercycler Nexus X2 under the following conditions: 95°C for 2 min; 35 cycles of 95°C for 15 s, 50°C for 15 s, 72°C for 20 s; 72°C for 2 min; and a holding temperature of 10°C. PCR products were observed via gel electrophoresis, and the DNA quality of each extract was quantified using a NanoDrop One Spectrophotometer.

Following verification of extracts, 50 µL of each extraction was sent to Zymo Research Corporation (Irvine, California, USA) for next‐generation sequencing (Illumina NextSeq) of the V4 region of the 16S rRNA gene using primers custom designed by Zymo. Returned sequences were processed in the mothur software package (v.1.43.0; Schloss et al. 2009) following the pipeline described in Erwin et al. (2017), with sequences aligned to the SILVA taxonomy database (v132.V4; full bioinformatics pipeline details in Supporting Information S1: Table S1). Rare operational taxonomic units (OTUs; ≤ 1 count) were removed and sequences were subsampled to the lowest sampling depth across all samples (*n* = 159,958). Updated microbial taxa nomenclature (Oren et al. 2023) has been used throughout this manuscript, and the presence of *Synechocystis* (OTU#2) confirmed via NCBI BLASTn searches (100% nucleotide sequence match to GenBank accession AB011380). Sequences have been deposited in NCBI SRA (PRJNA1138748).

### 2.3 Alpha‐ and Beta‐Diversity

Metrics of alpha‐diversity (Shannon's H’ diversity, OTU richness, and Pielou's evenness) were calculated for each sample in RStudio (2023.12.0 + 369; R version 4.3.2) and compared across samples (control, SW, SW + M, and ambient seawater) using the non‐parametric Kruskal–Wallis rank sum test using the “stats” package. Pairwise analyses were conducted using the Dunn test with Holm‐adjusted *p*‐values in the “FSA” package. Alpha‐diversity trends were visualized in boxplots using the “ggplot2” package.

The beta‐diversity metric Bray‐Curtis similarity was calculated for each sample and statistically compared across sample types with permutational analyses of variance (PERMANOVAs) in PRIMER 7 (version 7.0.21 from PRIMER‐e). To account for the small sample size of this study, primary test and pairwise PERMANOVAs were conducted using unrestricted permutations of raw data and *p*‐values determined using Monte Carlo tests. Data were visualized in a non‐metric multidimensional scaling (nMDS) plot.

### 2.4 OTU‐Level Analyses

An OTU‐level analysis was performed in MicrobiomeAnalyst 2.0 software, maintained by the Xia Lab (Chong et al. 2020), using the OTU table and consensus taxonomy file output by mothur with SILVA taxonomy labels. To identify the OTUs that were significantly different between sample pairs, the “multiple linear regression with covariate adjustment” was run in MicrobiomeAnalyst 2.0 using the linear model option.

## 3 Results

### 3.1 Amplicon Sequencing and Microbiome Composition

Of the 3,493,755 raw sequences generated, a total of 1,919,496 high‐quality sequences remained after processing in mothur (Supporting Information S1: Table S1) from all sample types (control, SW, SW + M, and ambient seawater), and clustered into 6612 OTUs based on 97% sequence similarity. All samples were dominated by the domain Bacteria, which was represented by 98.47% of all OTUs, with the domain Archaea only represented by 1.53% of OTUs (Supporting Information S1: Figure S2). At the phylum level, Pseudomonadota was the most abundant taxa across sample types, followed by Cyanobacteria (Figure 1). Alphaproteobacteria, Oxyphotobacteria, and Gammaproteobacteria were the most abundant classes across samples (Supporting Information S1: Figure S3), with more variation among samples evident at the order, family, and genus levels.

**Figure 2:**
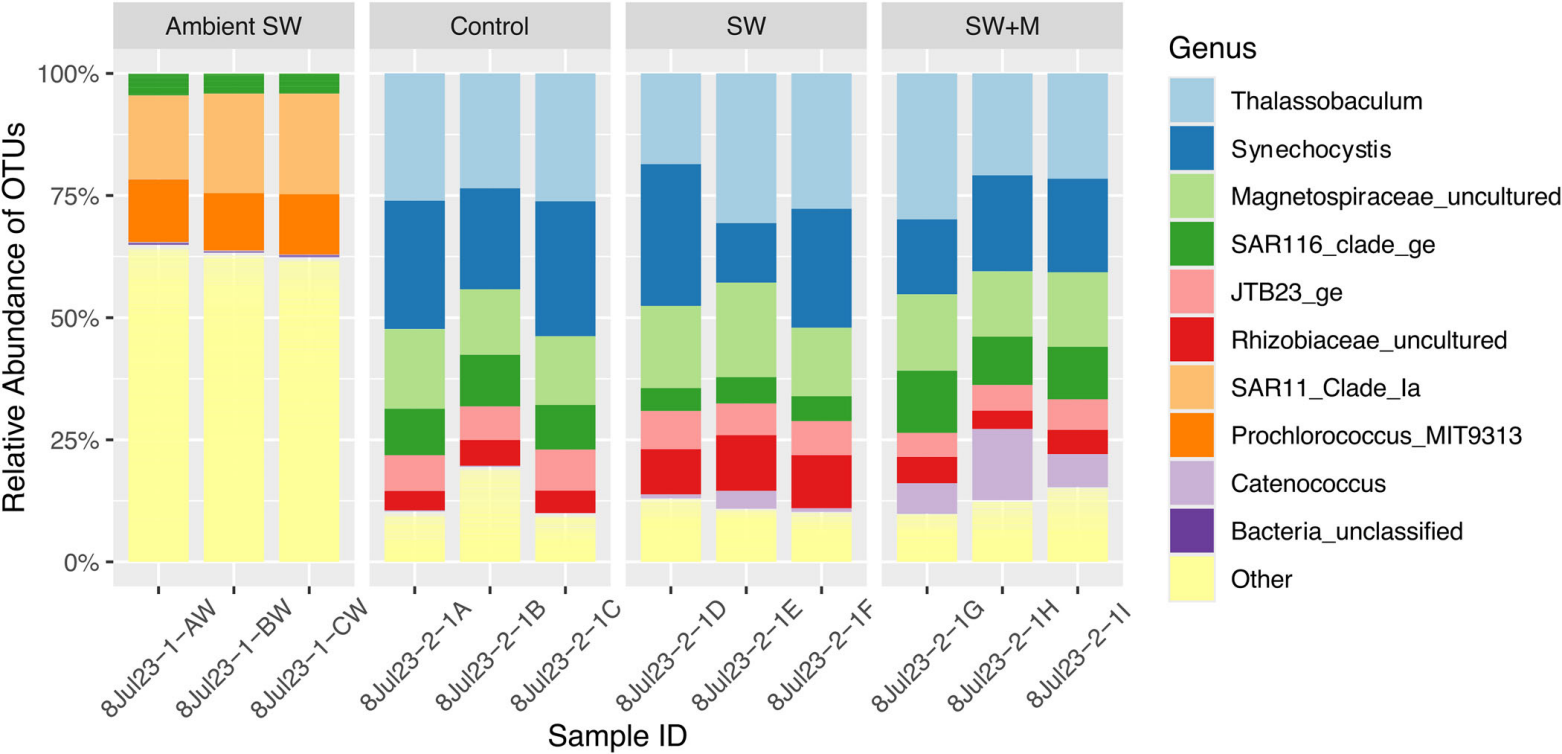
Genus‐level composition of microbial communities in *T. solidum* (Control, SW, SW + M) and ambient seawater (Ambient SW), showing the relative abundance of the top nine most abundant taxa. The “Other” category represents all other genera. Ascidian samples are grouped by preservation treatment: immediate (Control), 3‐h delay in seawater only (SW), and 3‐h delay in seawater and menthol (SW + M).

All ascidian microbiome samples were compositionally distinct from ambient seawater and impacts of delayed preservation were restricted to a few specific taxa. At the ordinal level, ambient seawater samples were dominated by members of the SAR11 clade and Synechococcales, which were significantly less abundant (ANOVA; all *p* < 0.001) than ascidian samples (Supporting Information S1: Table S2 and Figure S4). Meanwhile ascidian samples were dominated by orders Thalassobaculales, Nostocales, and Rhodospirillales, which were all significantly less abundant in ambient seawater (ANOVA; all *p* < 0.05; Supporting Information S1: Table S2 and Figure S4). Within ascidian samples, the control (immediate preservation) and delayed preservation with menthol treatment (SW + M) samples had significantly higher abundances (ANOVA; *p* < 0.01) of Puniceispirillales compared to the delayed preservation (SW) group, which exhibited a significantly higher abundance (ANOVA; *p* < 0.01) of Rhizobiales (Supporting Information S1: Table S2 and Figure S4). Vibrionales was significantly more abundant (ANOVA; *p* < 0.05) in the delayed preservation with menthol (SW + M) than the control, but not the delayed preservation without menthol (SW; Supporting Information S1: Table S2 and Figure S4). The most abundant microbial genera across all ascidian treatments were *Thalassobaculum*, *Synechocystis*, and an uncultured member of Magnetospiraceae (Figure 2 and Supporting Information S1: Figure S5). It is important to note that the representative sequences for *Synechocystis* were initially misidentified as *Prochloron‐P1‐Palau* due to the absence of a reference sequence for *Synechocystis trididemni* (GenBank accession AB011380) in the mothur‐formatted SILVA 132 database. Finally, the genus *Catenococcus* was significantly less abundant (ANOVA; *p* < 0.05) in the control samples compared to the samples from the SW + M treatment (Supporting Information S1: Table S2; Figure 2).

### 3.2 Alpha‐ and Beta‐Diversity

**Figure 3:**
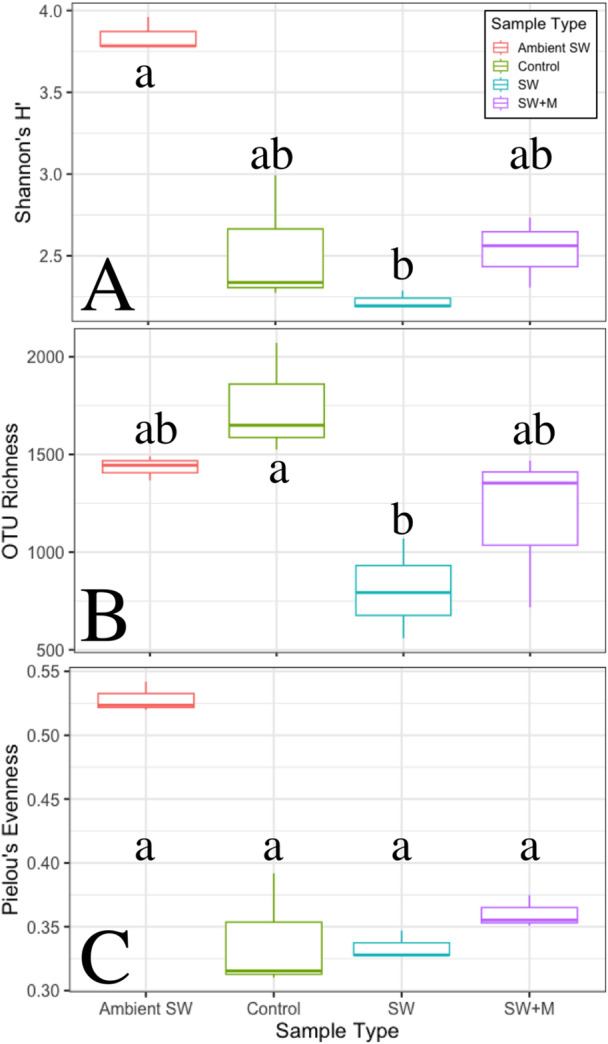
Boxplots of Shannon's H’ diversity (A), OTU richness (B), and Pielou's evenness (C) of microbial communities in *T. solidum* (Control, SW, SW + M) and ambient seawater (Ambient SW, red). Ascidian samples are grouped by preservation treatment: immediate (Control, green), 3‐h delay in seawater only (SW, blue), and 3‐h delay in seawater and menthol (SW + M, purple). Letters indicate the samples with significantly different alpha‐diversity metrics, as calculated by a pairwise Dunn test with Holm‐adjusted *p*‐values.

There were significant differences in Shannon's H’ diversity (Kruskal–Wallis; *χ* ^2^ = 8.744; *p* < 0.05) and richness (*χ* ^2^ = 8.744; *p* < 0.05) across samples, but not for Pielou's evenness (Table 1 and Supporting Information S1: Table S3). Pairwise analyses indicated that the ambient seawater samples were significantly more diverse (Pairwise Dunn; *z* = 2.944: Holm‐adjusted *p* < 0.05) than ascidian samples held in seawater for 3 h (SW; Figure 3A), and the control group had a microbiome with significantly greater richness (*z* = 2.831; *p* < 0.05) than the group held in seawater for 3 h (SW; Figure 3B). All other pairwise analyses for Shannon's H’ diversity, OTU richness, and Pielou's evenness were nonsignificant (Table 1; Figure 3A–C).

**Table 1:** Alpha‐diversity statistical analyses of microbial communities in *T. solidum* (Control, SW, SW + M) and ambient seawater (Ambient SW).

	Diversity			Richness			Evenness		
Kruskal–Wallis	*χ* ^2^	*p*	—	*χ* ^2^	*p*	—	*χ* ^2^	*p*	—
	8.744	**0.033**		8.744	**0.033**		7.615	0.055	
*Pairwise Dunn*	*z*	*p*	*Holm*	*z*	*p*	*Holm*	*z*	*p*	*Holm*
Ambient SW × Control	1.698	0.089	0.447	−1.246	0.213	0.639	2.378	0.017	0.105
Ambient SW × SW	2.944	0.003	**0.019**	1.585	0.113	0.452	2.378	0.017	0.087
Control × SW	1.246	0.213	0.426	2.831	0.005	**0.028**	0.000	1.000	1.000
Ambient SW × SW+M	1.472	0.141	0.423	0.793	0.428	0.428	1.359	0.174	0.697
Control × SW+M	−0.226	0.821	0.821	2.038	0.042	0.208	−1.019	0.308	0.925
SW × SW+M	−1.472	0.141	0.564	−0.793	0.428	0.856	−1.019	0.308	0.616

*Note:* Ascidian samples are grouped by preservation treatment: immediate (Control), 3‐h delay in seawater only (SW), and 3‐h delay in seawater and menthol (SW + M). Main test Kruskal–Wallis *χ* ^2^ and *p*‐values (*p*) are reported for Shannon's H’ diversity, OTU richness, and Pielou's evenness. Pairwise Dunn test results for each metric show *z*‐scores (*z*), *p*‐values (*p*), and Holm‐adjusted *p*‐values (Holm). Bold text indicates significant results.

**Figure 4:**
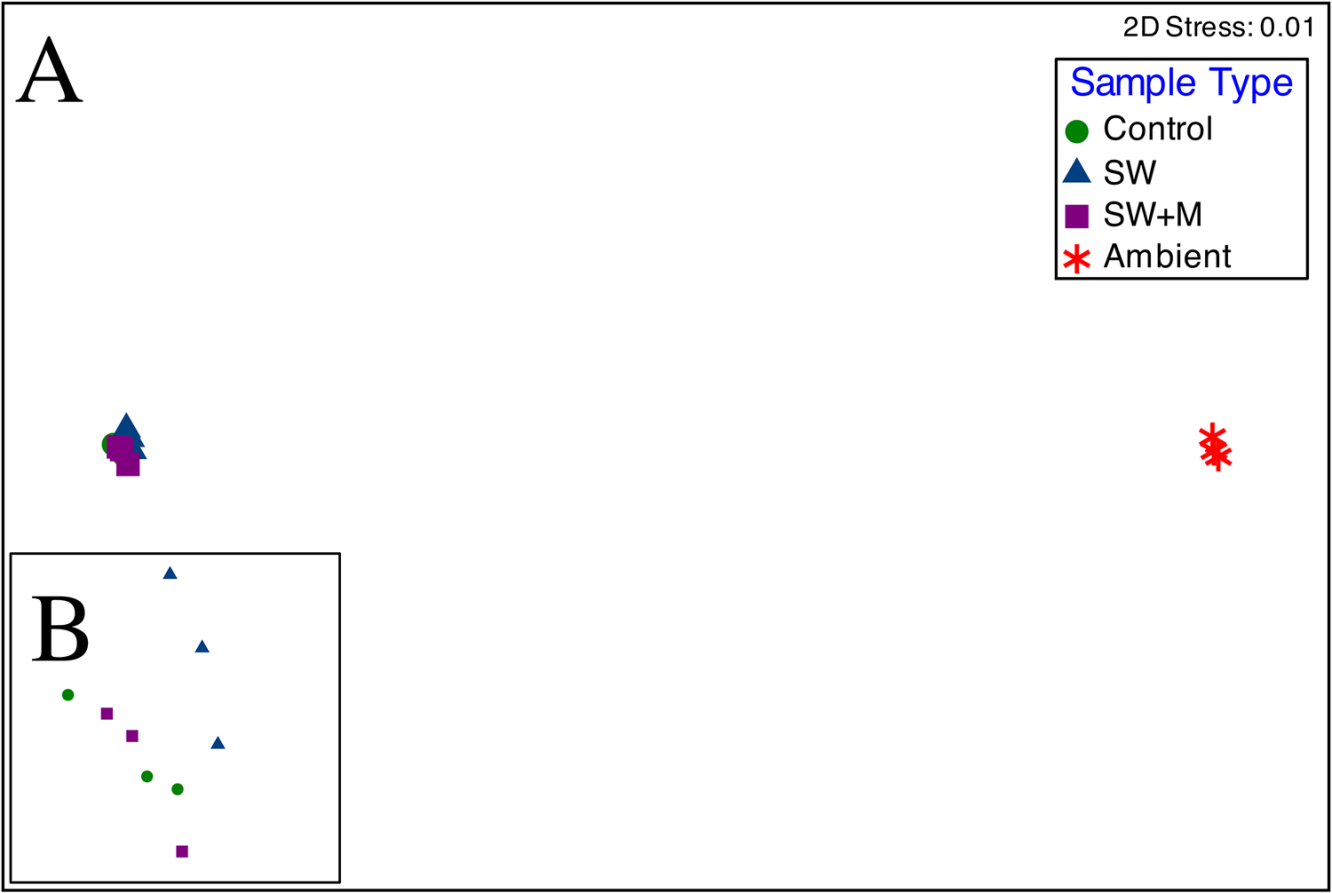
Nonmetric multidimensional scaling (nMDS) plot of microbial communities in *T. solidum* (Control, SW, SW + M) and ambient seawater (Ambient SW, red asterisks) based on Bray‐Curtis similarity (stress = 0.01) of relative abundance OTU data (A). Ascidian samples are grouped by preservation treatment: immediate (Control, green circles), 3‐h delay in seawater only (SW, blue downward triangle), and 3‐h delay in seawater and menthol (SW + M, purple upward triangle). Inset (B) shows a magnified view of ascidian samples.

Microbiome composition was significantly different across sample types (Bray‐Curtis similarity; PERMANOVA; pseudo‐*F* = 77.469: *p* < 0.001; Table 2) and sample type accounting for 83.3% of microbiome variation. Pairwise analyses revealed significant differences in microbiome structure between all ascidian samples and ambient seawater (Pairwise PERMANOVA; *p* < 0.01). Within ascidian samples, significant differences were detected between delayed preservation treatments (with and without menthol; *t* = 2.119; *p* < 0.05) but not between control and SW (*t* = 1.924; *p* = 0.054) and control and SW + M treatments (*t* = 1.940; *p* = 0.052; Table 2). The nMDS plot based on microbiome similarity (stress = 0.01) clustered the samples into two distinct groups: (1) the ambient seawater samples and (2) all ascidian samples (Figure 4A,B).

**Table 2:** Beta‐diversity statistical analyses of microbial communities in *T. solidum* (Control, SW, SW+M) and ambient seawater (Ambient SW).

	*Pseudo‐F*	*P(MC)*
*PERMANOVA*	77.469	**0.001**
*Pairwise PERMANOVA*	*t*	*P(MC)*
Control × SW	1.924	0.054
Control × SW+M	1.940	0.052
Control × Ambient SW	16.653	**0.001**
SW × SW+M	2.119	**0.034**
SW × Ambient SW	12.149	**0.001**
SW+M × Ambient SW	14.329	**0.001**

*Note:* Ascidian samples are grouped by preservation treatment: immediate (Control), 3‐h delay in seawater only (SW), and 3‐h delay in seawater and menthol (SW+M). Pseudo‐*F* values are reported for main test PERMANOVA and *t* values for pairwise PERMANOVA analyses, with Monte Carlo (MC) *p*‐values shown for both. Bold text indicates significant results.

### 3.3 OTU‐Level Analyses

Ascidian microbiomes were dominated by a small number of bacterial OTUs found in all treatments but absent or extremely rare in ambient seawater (Table 3). The 10 most abundant OTUs in control samples accounted for 89.4% of total sequences within the treatment type. These 10 OTUs similarly dominated the microbiomes of samples preserved 3 h after the controls, comprising 94.4% (without menthol; SW) and 91.5% (with menthol; SW + M) of all sequences. In contrast, these OTUs were extremely rare in ambient seawater, accounting for only 0.30% of bacterioplankton sequences. Two OTUs (*Thalassobaculum* OTU#1, *Synechocystis* OTU#2) comprised nearly half of all *T. solidum* microbiomes (42%–50%) and were absent in ambient seawater (Table 3).

**Table 3:** Taxonomy and relative abundance of the 10 most abundant OTUs in the microbiome of *T. solidum* by preservation treatment: immediate (Control), 3‐h delay in seawater only (SW), and 3‐h delay in seawater and menthol (SW + M).

				*Trididemnum solidum*		
OTU	Phylum	Lowest taxonomy	Seawater	Control	SW	SW + M
#1	Pseudomonadota (α)	Genus *Thalassobaculum*	0.00 ± 0.00	25.18 ± 0.87	25.62 ± 3.63	24.06 ± 2.88
#2	Cyanobacteria	Genus *Synechocystis*	0.00 ± 0.00	24.88 ± 2.11	21.84 ± 5.02	18.05 ± 1.35
#3	Pseudomonadota (α)	Family Magnetospiraceae	0.00 ± 0.00	14.57 ± 0.88	16.71 ± 1.54	14.73 ± 0.69
#4	Pseudomonadota (α)	Family SAR116	0.22 ± 0.00	9.72 ± 0.43	5.06 ± 0.21	11.11 ± 0.84
#7	Pseudomonadota (γ)	Order JTB23	0.00 ± 0.00	7.51 ± 0.46	7.09 ± 0.39	5.47 ± 0.39
#6	Pseudomonadota (α)	Family Rhizobiaceae	0.00 ± 0.00	4.65 ± 0.37	10.50 ± 0.63	4.72 ± 0.49
#10	Pseudomonadota (γ)	Genus *Catenococcus*	0.08 ± 0.00	0.05 ± 0.03	1.73 ± 0.96	9.11 ± 2.73
#11	Pseudomonadota (α)	Genus *Tistrella*	0.00 ± 0.00	1.36 ± 0.29	3.25 ± 0.79	2.23 ± 0.11
#13	Pseudomonadota (α)	Class Alphaproteobacteria	0.00 ± 0.00	0.84 ± 0.11	2.18 ± 0.16	0.85 ± 0.10
#19	Actinomycetota	Genus *Ilumatobacter*	0.00 ± 0.00	0.60 ± 0.16	0.39 ± 0.03	1.12 ± 0.23

*Note:* Phylum‐level classifications are shown, with the Pseudomonadota classes Alphaproteobacteria (α) and Gammaproteobacteria (γ) in parentheses, followed by lowest lowest‐level taxonomic affiliations of each dominant OTU. Average OTU relative abundance (± 1 standard error) in ambient seawater and ascidian treatments are reported.

Pairwise comparisons using multiple linear regression with covariate adjustment indicated that delayed preservation (SW and SW + M) treatments were most similar at the OTU‐level, with only 54 OTUs that were significantly different (0.82% of total OTUs; Supporting Information S1: Table S4). Between control and SW + M, only 65 OTUs (0.98% of total OTUs) were significantly different, while 122 OTUs (1.85% of total OTUs) were different between control and SW (Supporting Information S1: Table S4). All ascidian treatments were clearly differentiated from ambient seawater, where the number of significantly differential OTUs ranged from 575 to 795 (8.70%–12.02% of total OTUs; Supporting Information S1: Table S4).

**Figure 5:**
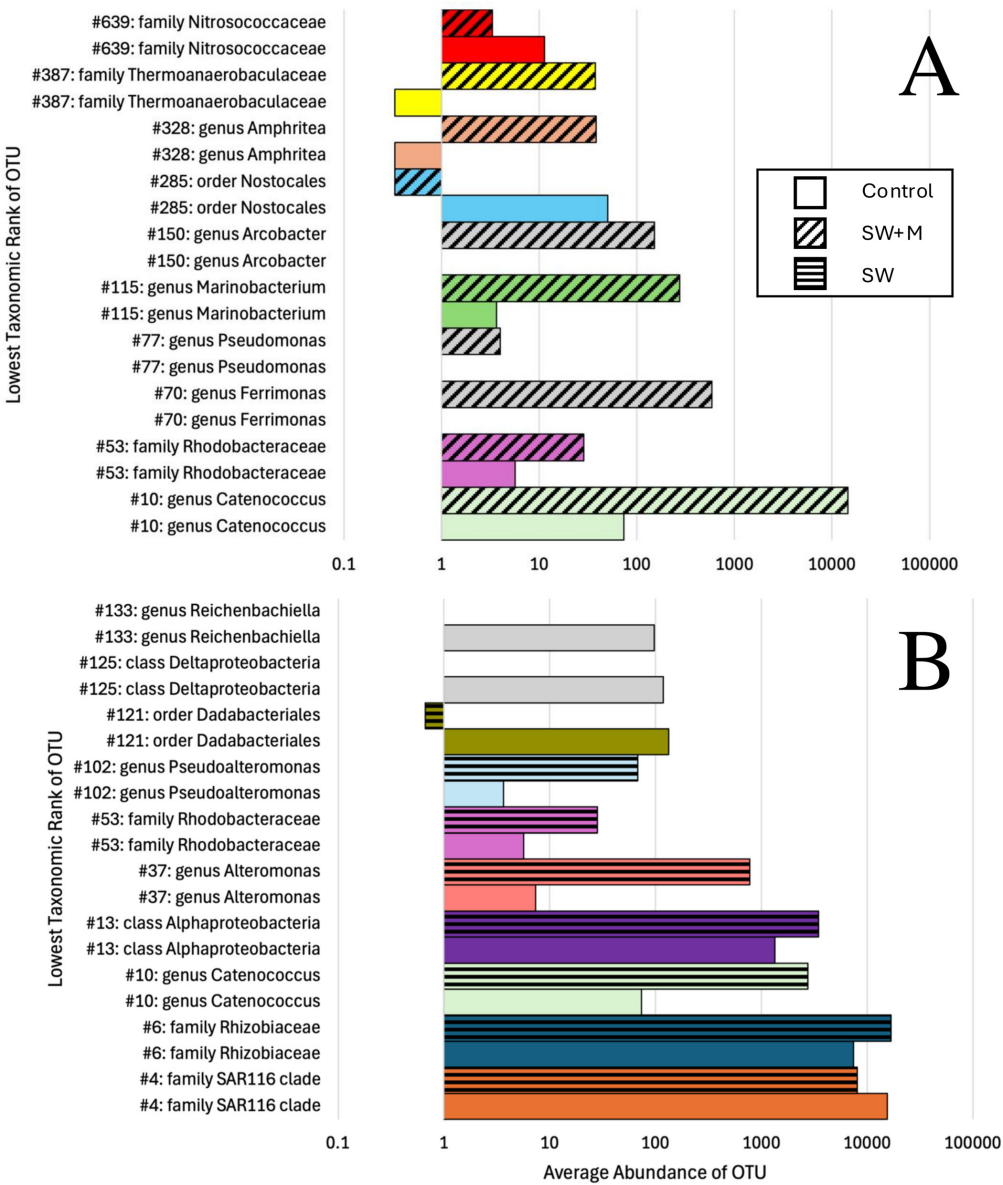
Bar plots of the 10 most abundant OTUs in the *T. solidum* microbiome that differed significantly between immediately preserved samples (Control, solid colors) and delayed preservation with (SW + M, diagonal stripes, A), and without menthol (SW, horizontal stripes, B). Average abundance values are shown on the x‐axis, which is formatted in a “logarithmic scale.” OTU number and lowest taxonomic rank are listed on the y‐axis. Gray bars indicate OTUs only present in one treatment.

Analysis of the top 10 most abundant differential OTUs across all samples for pairwise comparisons between treatments (Supporting Information S1: Table S5) revealed fine‐scale patterns of taxonomic shifts in OTU membership. Between the control samples and the SW + M samples, the most abundant OTUs were a member of gammaproteobacteria, *Catenococcus* (OTU#10), and alphaproteobacteria belonging to the family Rhodobacteraceae (OTU#53), both of which were more abundant in SW + M (Figure 5A). There were three OTUs (#s 70, 77, 150) that were absent in the control samples, but present in SW + M samples, which belonged to the genera *Ferrimonas*, *Pseudomonas*, and *Arcobacter,* respectively (Figure 5A). Overall, the average abundance of significantly differential OTUs between control and SW samples was higher than control and SW + M samples (Figure 5). The most abundant of these OTUs included the same *Catenococcus* OTU above (#10) and three alphaproteobacteria: OTU#4 belonging to the SAR116 family, OTU#6 belonging to family Rhizobiaceae, and OTU#13, an unclassified alphaproteobacterium (Figure 5B). Two OTUs (#125, 133) were present in the control treatment but absent in the SW treatment, one belonging to class Deltaproteobacteria and the other in genus *Reichenbachiella* (Figure 5B).

## 4 Discussion

The influence of delayed preservation time and menthol addition on the microbiome of the colonial ascidian *Trididemnum solidum* was investigated for samples immediately preserved in ethanol (control), after a 3‐h preservation delay (SW), and after a 3‐h preservation delay with menthol crystals (SW + M). All samples hosted microbiomes that were clearly differentiated from microbial communities in ambient seawater and were dominated by the same 10 bacterial OTUs. Preservation delays changed the microbiome of the host species in terms of richness and OTU‐level composition though these differences were restricted to rare taxa and were much less pronounced than comparisons to free‐living seawater communities. Furthermore, the addition of menthol crystals (SW + M) appeared to mitigate the decreases in richness and compositional shifts that were observed in samples held in only ambient seawater (SW). Together, these results indicated that delayed preservation has minimal impact on ascidian microbiome characterization and that the common practice of menthol treatment before preservation counteracts microbiome shifts associated with processing delays.

Dominant microbial taxa in *T. solidum* were largely unaffected by preservation delays and menthol treatment, exhibiting stability despite changing environmental conditions, and no significant differences in community‐level microbiome composition occurred between delayed preservation treatments and control treatments for ascidians. Consistent with these findings, previous studies have reported high stability and specificity of colonial ascidian microbiomes (Erwin et al. 2014; López‐Legentil et al. 2015, 2023) and shown no significant differences in microbiome richness and composition following 3 days of starvation conditions (Dishaw et al. 2014). Dominant symbionts in other invertebrate microbiomes similarly remain unchanged despite transfer to aquaria, temperature stress, starvation, and antibiotic treatment (Pita et al. 2013; Schmittmann et al. 2022). This stability may be due to distinct microenvironments inside the host that remain unchanged during environmental changes and mutualistic relationships between the host and symbiont. Notably, some of the dominant taxa in *T. solidum* may similarly play important metabolic roles in the ascidian holobiont, including photosynthetic carbon‐fixation (*Synechocystis*, Olson 1986) and secondary metabolite synthesis (*Tistrella*, Xu et al. 2012). In contrast, rare components of the ascidian microbiomes may play redundant roles or act as commensal symbionts, resulting in greater spatiotemporal fluctuations as conditions change.

Menthol has documented antimicrobial effects (İşcan et al. 2002; Trombetta et al. 2005) and appears to mitigate ascidian microbiome changes during short‐term delays in preservation. Indeed, no significant differences in microbial diversity or composition were observed between the control and menthol (SW+M) treatments, possibly due to aqueous menthol disproportionally disrupting the plasma membranes of bacterioplankton in the holding tank compared to symbiotic bacteria within the ascidian tissue. Cyanobacterial cells associated with *Trididemnum* ascidians have been imaged inside the cellulose tunic (Hirose and Hirose 2007; López‐Legentil et al. 2011) and among the calcareous spicules embedded in the tunic (Olson 1986) of their hosts. The tunic provides physical protection and houses a diverse collection of free cells that may have evolved from a single cell type, the phagocyte, which plays a role in the ascidian immune response (Hirose 2009). Given this functionality, symbiotic bacteria within the tunic of *T. solidum* may be less exposed to the antimicrobial effects of menthol that impede biological activity of the microbes in the surrounding seawater. Additionally, the relaxing effect of menthol prevents the ascidian host from actively pumping seawater, which may lessen the interaction of microbial symbionts with bacterioplankton within the surrounding seawater. In the treatments where ascidian samples were held in seawater without menthol for 3 h, however, interactions between ascidian holobionts and bacterioplankton could have resulted in microbiome shifts due to the lack of menthol (an antimicrobial and relaxing substance). For example, OTU#37 (*Alteromonas*) and #102 (*Pseudoalteromonas*) represent common bacterioplankton taxa found in ambient seawater and were more abundant in samples held in seawater without menthol (SW) than those with menthol (SW + M) or immediately preserved (control). Such colonization of *T. solidum* by bacterioplankton during delayed preservation without the addition of an antimicrobial substance could be influenced by changes in limiting resource availability, pressures from external predators, and competition between bacterial taxa upon transfer from the natural habitat to holding tanks, all of which are key to consider within microbial food webs (Våge et al. 2018). Our study did not directly test the effects of menthol on bacterioplankton, thus future experiments characterizing seawater bacteria in holding tanks with and without menthol crystals are required to further investigate these potential mechanisms.

In addition to colonization by bacterioplankton, minor shifts in composition during preservation delay may result from changes in the relative abundance of symbiont taxa within host tissues. For example, a member of family Rhizobiaceae (OTU#6) had an average relative abundance of 4.65 ± 0.37% in the control and 4.72 ± 0.49% in the menthol treatments (SW + M) but had an average relative abundance of 10.50 ± 0.63% in the SW treatment. This OTU was absent in the ambient seawater samples, suggesting that the bloom occurred within the ascidian microbiome. Similarly, *Catenococcus* (OTU#10) had an average relative abundance of 0.05 ± 0.03% in the control samples and was significantly more abundant in both SW (1.73 ± 0.96%) and SW + M (9.11 ± 2.73%) treatments. However, this OTU was present in the ambient seawater samples at an abundance comparable to that of the control (0.08 ± 0.004), so *Catenococcus* could have proliferated from either within the pre‐existing ascidian microbiome or following transfer from seawater. OTU#10 had a 100% match with *Vibrio alginolyticus* isolated from a marine sponge (*Rhabdastrella globostellata*; Acc. num. MW255268.1). Members of *Catenococcus* have been found as infective agents in giant clams (Guibert et al. 2020) and stony corals (Fifer et al. 2022), and *Vibrio alginolyticus* is a documented pathogen of amphioxus (Zou et al. 2016) and sea cucumbers (Fahmy and Hamed 2022). Thus, a delay in preservation may trigger the spread of potentially pathogenic bacteria in *T. solidum*, which may have a weakened immune response when held in ambient seawater for hours, while menthol appears to reduce the scale of blooms.

Immediate preservation of samples for microbiome analysis is always recommended; however, delays in preservation of marine invertebrates are common in the field and for specific taxa requiring pre‐preservation treatments (e.g., menthol relaxation) for species identification. Our study shows that short‐term (3 h) delays in preservation did not impact dominant symbiont taxa or significantly alter community‐level microbiome composition. Fine‐scale changes in primarily rare taxa were detected at the OTU‐level, with decreased microbial richness and shifts in rare OTU composition occurring after a 3‐h preservation delay. The addition of menthol appeared to counteract the effect of delayed preservation, exhibiting the same microbiome richness as immediately preserved *T. solidum* samples. This work may inform future studies when a delay in preservation is necessary, such as for the depuration process when invertebrate hosts need to be kept in aquaria (e.g., Griffin et al. 2023). As a single colony of *T. solidum* was used in this study, future work should address any interspecific variation in treatment responses, particularly between solitary and colonial ascidian species. The effects of delayed preservation on microbial gene expression should also be considered, as metatranscriptomic responses to preservation methods have yet to be explored and may provide further insight into host‐symbiont dynamics in invertebrate holobionts.

## Author Contributions

**Brenna Hutchings:** conceptualization (equal), formal analysis (lead), writing ‐ original draft (lead), writing – review and editing (lead). **Susanna López‐Legentil:** conceptualization (equal), formal analysis (supporting), funding acquisition (equal), writing – original draft (supporting), writing – review and editing (supporting). **Lauren M. Stefaniak:** Funding acquisition (equal), writing – review and editing (supporting). **Marie Nydam:** funding acquisition (equal), writing – review and editing (supporting). **Patrick M. Erwin:** conceptualization (equal), formal analysis (supporting), funding acquisition (equal), writing – original draft (supporting), writing – review and editing (supporting).

## Ethics Statement

The authors have nothing to report.

## Conflicts of Interest

The authors declare no conflicts of interest.

## Supporting information

Hutchings etal MO REV Supplemental.

## Data Availability

The data that support the findings of this study are openly available in NCBI Sequence Read Archive at https://www.ncbi.nlm.nih.gov/sra, reference number PRJNA1138748.
