# Supplementary material for: Microbial Distortion? Impacts of Delayed Preservation on Microbiome Diversity and Composition in a Marine Invertebrate
Source: Microbiologyopen. 2025 May 15;14(2):e70019. doi: 10.1002/mbo3.70019 (PMC12081327; doi:10.1002/mbo3.70019)
Supplement: Supplementary file 1 — Hutchings etal MO REV Supplemental. [file MBO3-14-e70019-s001.docx]

**Microbial distortion? Impacts of delayed preservation on microbiome diversity and composition in a marine invertebrate**

Brenna Hutchings^1^ ⋅ Susanna López-Legentil^1^ ⋅ Lauren Stefaniak^2^ ⋅ Marie Nydam^3^ ⋅ Patrick M. Erwin^1^

^1^*Department of Biology & Marine Biology, and Center for Marine Science, University of North Carolina Wilmington, 5600 Marvin K. Moss Lane, Wilmington NC 28409, United States of America*

^2^*Department of Marine Science, Coastal Carolina University, 100 Chanticleer Dr. E., Conway SC 29528, United States of America*

^3^*Life Sciences Concentration, Soka University of America, 1 University Drive, Aliso Viejo CA 92656, United States of America*

**Supplementary Materials**
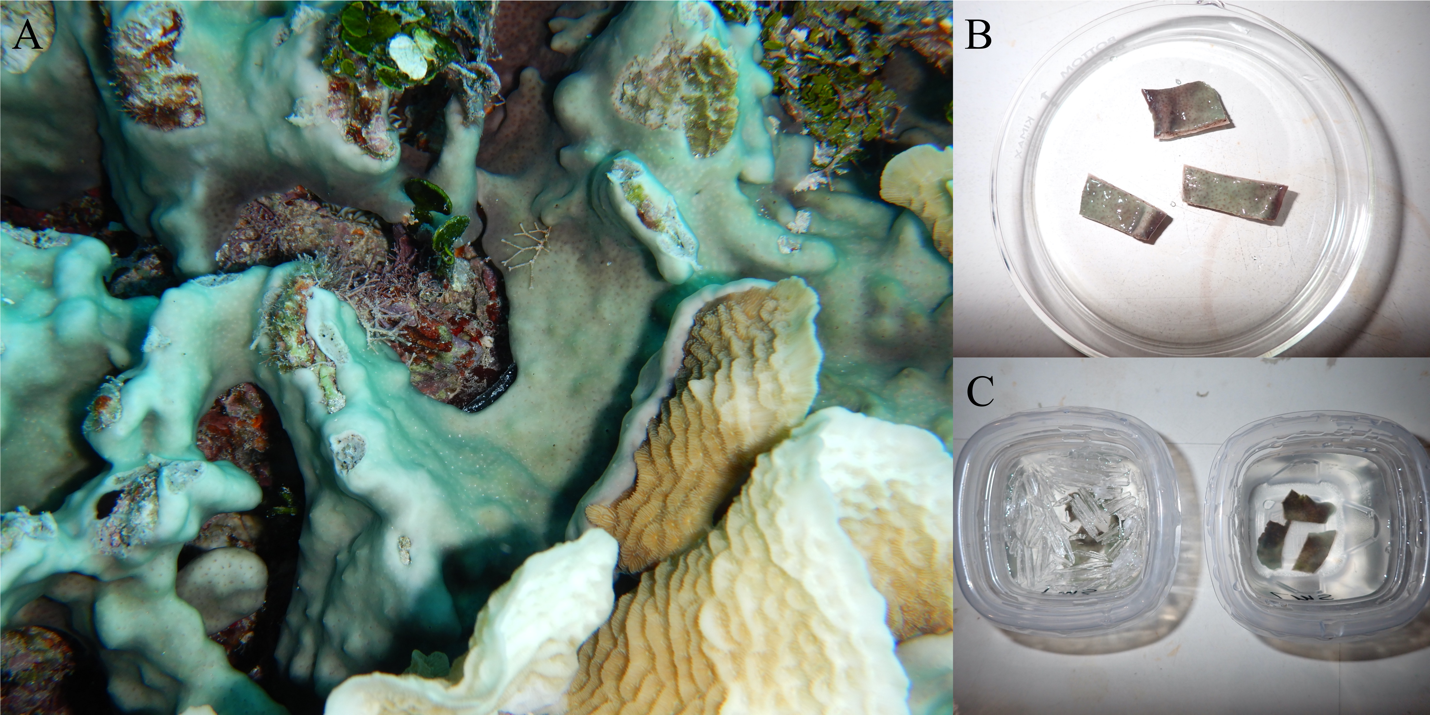


**Figure S1** *Trididemnum solidum* colony (green-grey in color, overgrowing coral) in situ (A); replicates for immediate preservation in ethanol (B); replicates held for 3 hours in ambient seawater prior to preservation, with (left) and without (right) menthol crystals (C).


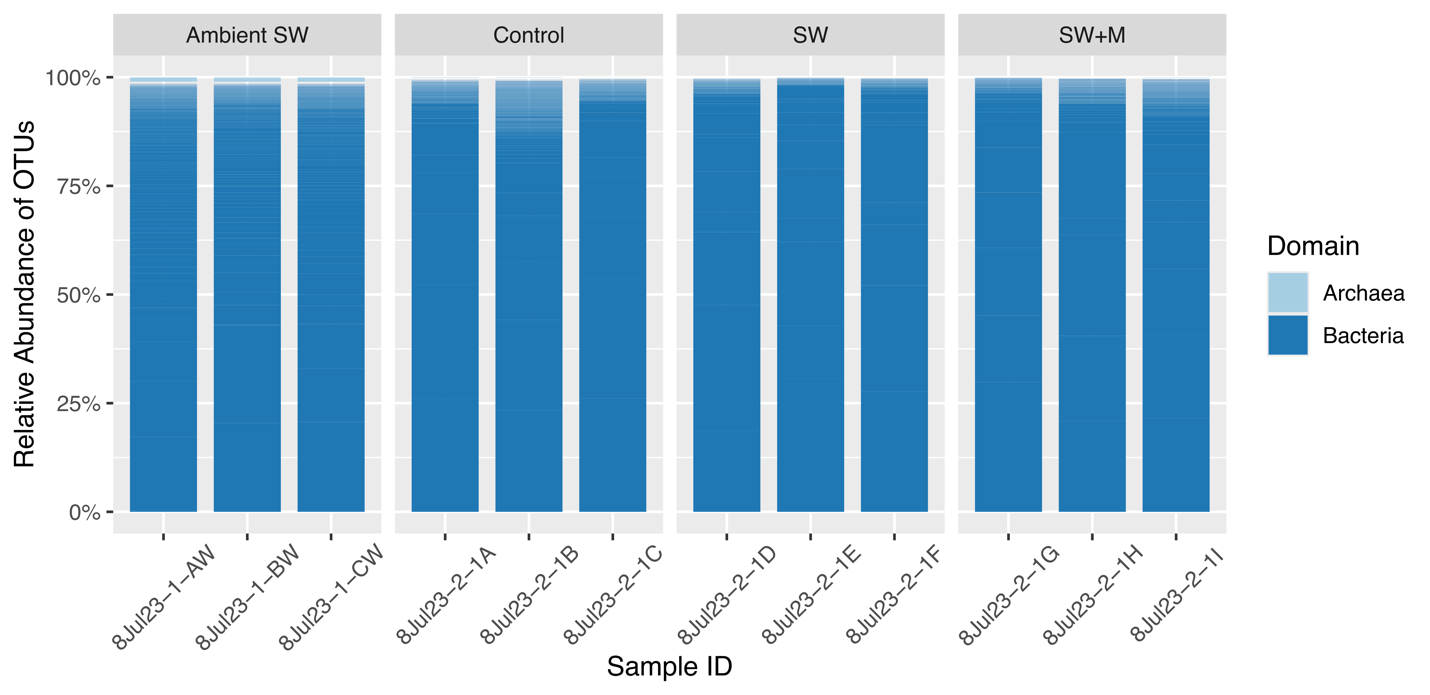


**Figure S2** Domain-level composition of microbial communities in *T. solidum* (Control, SW, SW+M) and ambient seawater (Ambient SW), showing the relative abundance of the top nine most abundant taxa. Ascidian samples are grouped by preservation treatment: immediate (Control), 3-hour delay in seawater only (SW), and 3-hour delay in seawater and menthol (SW+M).


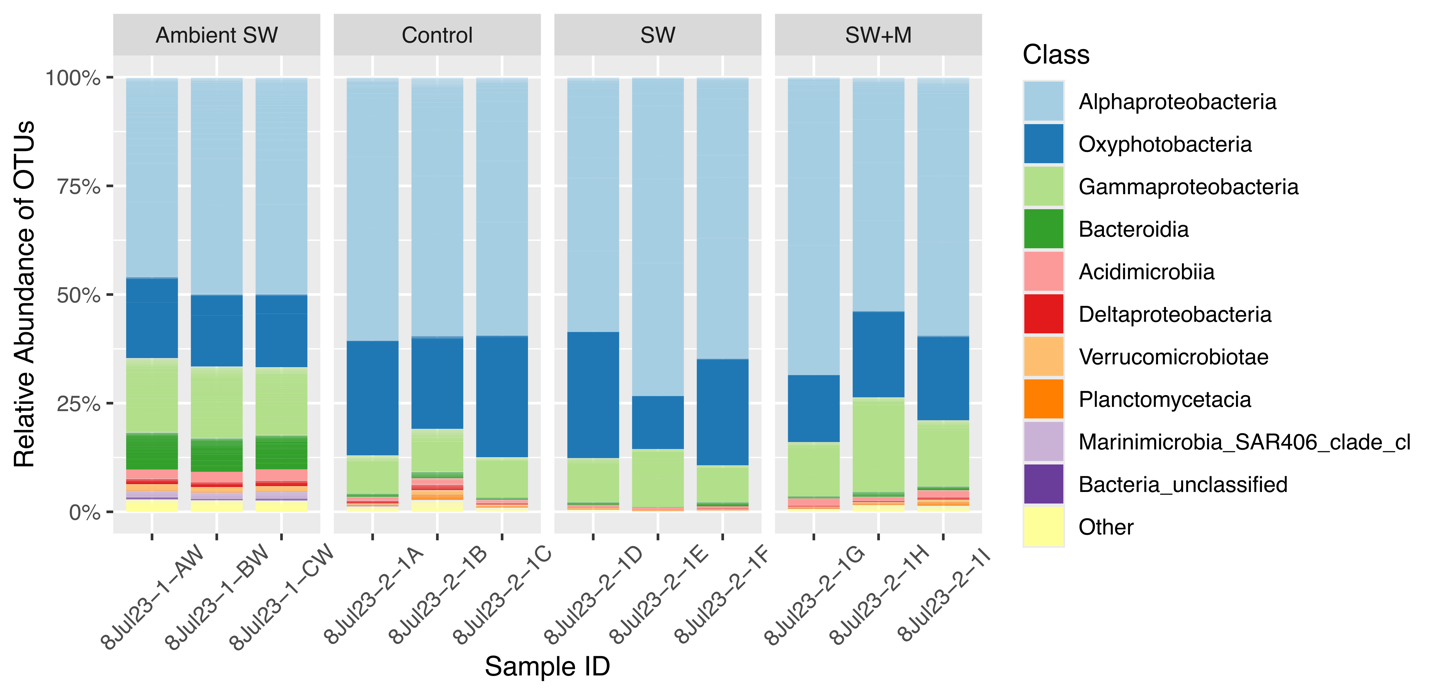


**Figure S3** Class-level composition of microbial communities in *T. solidum* (Control, SW, SW+M) and ambient seawater (Ambient SW), showing the relative abundance of the top nine most abundant taxa. The “Other” category represents all other classes. Ascidian samples are grouped by preservation treatment: immediate (Control), 3-hour delay in seawater only (SW), and 3-hour delay in seawater and menthol (SW+M).

**
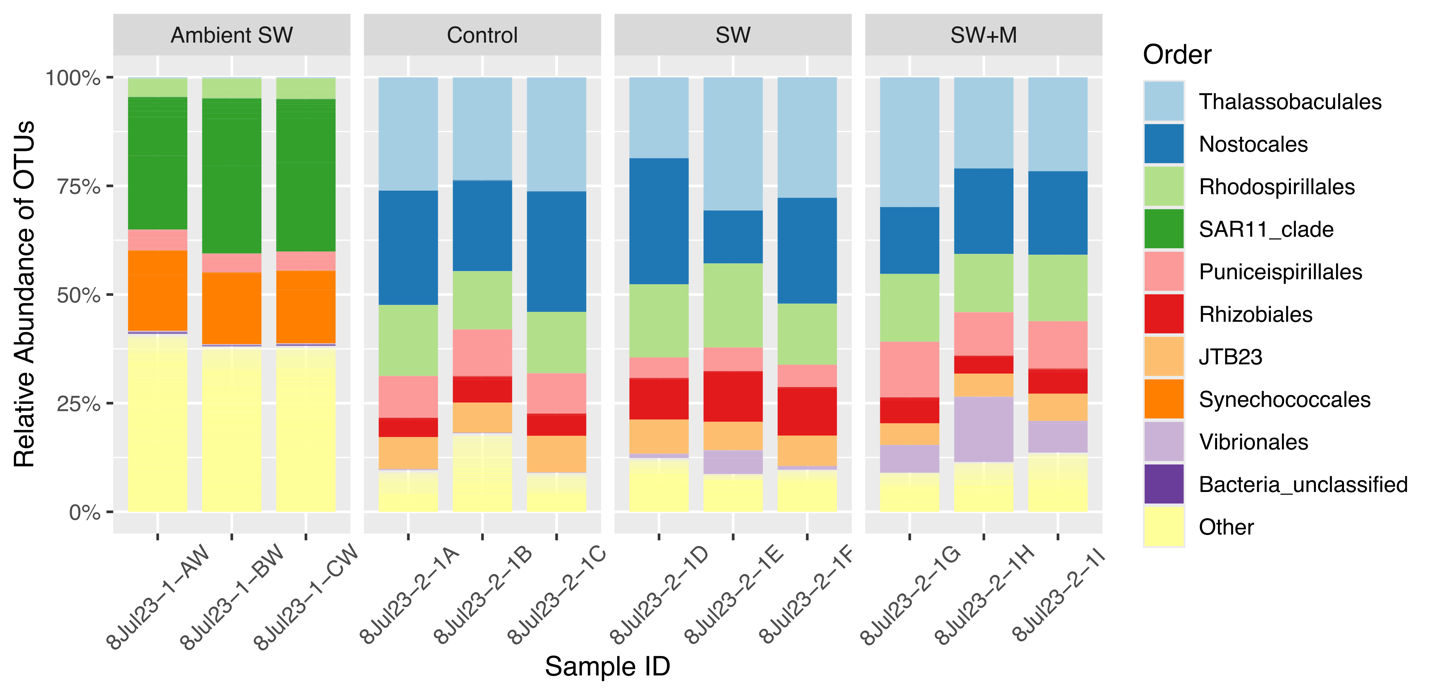
**

**Figure S4** Order-level composition of microbial communities in *T. solidum* (Control, SW, SW+M) and ambient seawater (Ambient SW), showing the relative abundance of the top nine most abundant taxa. The “Other” category represents all other orders. Ascidian samples are grouped by preservation treatment: immediate (Control), 3-hour delay in seawater only (SW), and 3-hour delay in seawater and menthol (SW+M).

**
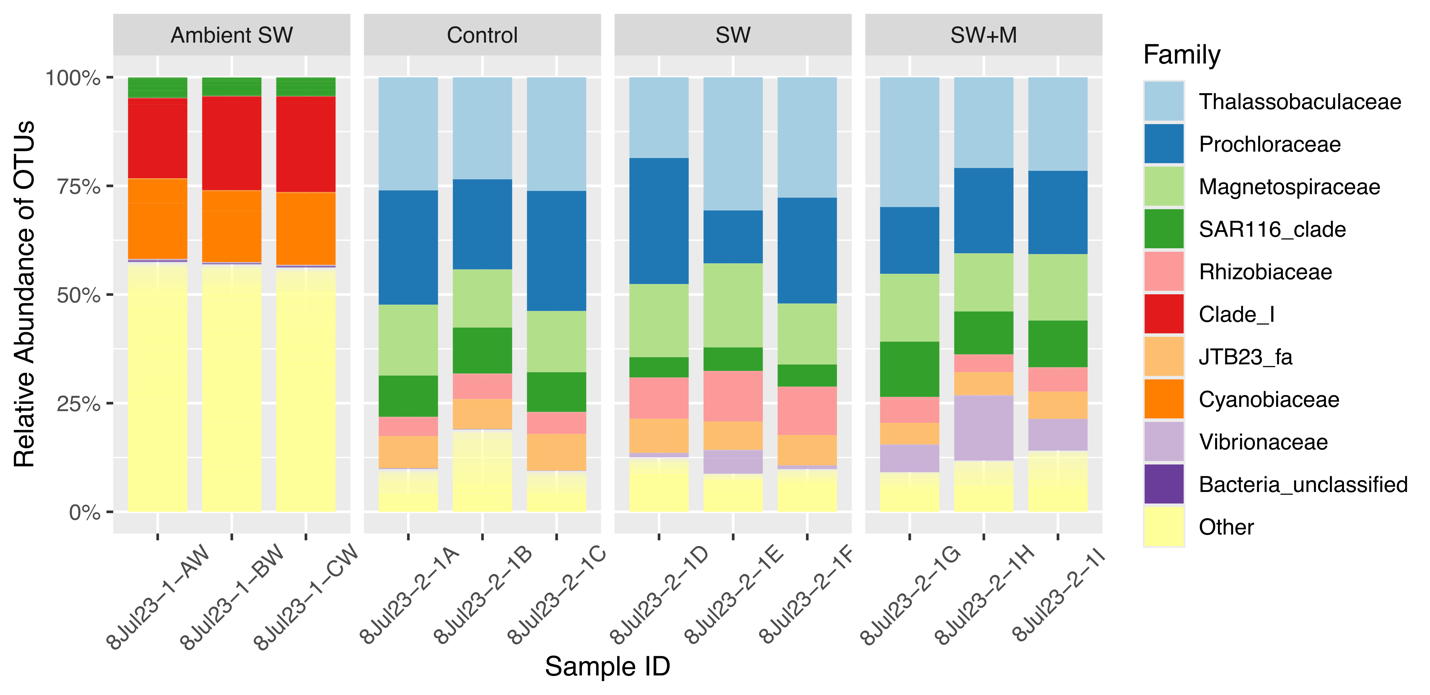
**

**Figure S5** Family-level composition of microbial communities in *T. solidum* (Control, SW, SW+M) and ambient seawater (Ambient SW), showing the relative abundance of the top nine most abundant taxa. The “Other” category represents all other families. Ascidian samples are grouped by preservation treatment: immediate (Control), 3-hour delay in seawater only (SW), and 3-hour delay in seawater and menthol (SW+M).

**Table S1** Mothur (v.1.43.0) bioinformatics pipeline followed for processing raw sequences.

| Command | Input File Type | Settings |
| --- | --- | --- |
| make.file | .gz |  |
| make.contigs | .fastq | trimoverlap=T, oligos=515f-806r.oligos, pdiffs=4, processors=8 |
| summary.seqs | .fasta |  |
| screen.seqs | .fasta, .groups | maxambig=0, maxlength=300, minlength=200, maxhomop=8 |
| summary.seqs | .fasta |  |
| unique.seqs | .fasta |  |
| align.seqs | .fasta | reference=silva.nr_v132.V4.align, processors=4 |
| summary.seqs | .fasta, .names |  |
| screen.seqs | .fasta, .groups, .names | start=1967, end=11549 |
| summary.seqs | .fasta, .names |  |
| filter.seqs | .fasta | vertical=T, trump=. |
| summary.seqs | .fasta, .names |  |
| pre.cluster | .fasta, .names, .groups | diffs=2 |
| summary.seqs | .fasta, .count_table |  |
| chimera.uchime | .fasta, .count_table | dereplicate=t, reference=self |
| remove.seqs | .fasta, .count_table, .accnos |  |
| summary.seqs | .fasta, .count_table |  |
| classify.seqs | .fasta, .count_table | reference= silva.nr_v132.V4.align, taxonomy=silva.nr_v132.tax, cutoff=60 |
| remove.lineage | .fasta, .count_table, .taxonomy | taxon=Chloroplast-Mitochondria-Eukaryota-unknown |
| summary.seqs | .fasta, .count_table |  |
| filter.seqs | .fasta | vertical=T, trump=. |
| rename.file | .fasta, .count_table, .taxonomy |  |
| dist.seqs | .fasta | cutoff=0.03, processors=4 |
| cluster | .dist, .count_table | cutoff=0.03 |
| remove.rare | .list, .count_table | nseqs=1, label=0.03 |
| classify.otu | .list, .count_table, .taxonomy | label=0.03 |
| get.oturep | .fasta, .count_table, .list | method=abundance |
| make.shared | .list, .count_table |  |
| count.groups | .count_table |  |
| sub.sample | .list, .count_table | persample=t, size=159,958 |
| count.groups | .count_table |  |
| list.otulabels | .list |  |
| get.otulabels | .otulabels, .cons.taxonomy |  |
| make.shared | .list, .count_table |  |

**Table S2** Identity of bacterial taxa of interest from stacked barplots, F statistic, and p-value indicating significant differences in relative abundance between sample type using ANOVA. Significant values in bold.

| Comparison | Identity | F | p-value |
| --- | --- | --- | --- |
| Control x SW | Order SAR 11 Clade | 2.163 | 0.215 |
| Control x SW+M |  | 4.500 | 0.101 |
| SW x SW+M |  | 1.385 | 0.305 |
| Ambient SW x Control |  | 424.569 | **3.277x10^-5^** |
| Ambient SW x SW |  | 424.490 | **3.278x10^-5^** |
| Ambient SW x SW+M |  | 424.553 | **3.278x10^-5^** |
| Control x SW | Order Synechococcales | 2.650 | 0.179 |
| Control x SW+M |  | 0.454 | 0.537 |
| SW x SW+M |  | 3.462 | 0.136 |
| Ambient SW x Control |  | 729.244 | **1.118x10^-5^** |
| Ambient SW x SW |  | 730.592 | **1.114x10^-5^** |
| Ambient SW x SW+M |  | 729.826 | **1.116x10^-5^** |
| Control x SW | Order Thalassobaculales | 0.010 | 0.926 |
| Control x SW+M |  | 0.150 | 0.718 |
| SW x SW+M |  | 0.109 | 0.758 |
| Ambient SW x Control |  | 880.766 | **7.676x10^-6^** |
| Ambient SW x SW |  | 49.363 | **0.002** |
| Ambient SW x SW+M |  | 69.528 | **0.001** |
| Control x SW | Order Nostocales | 0.343 | 0.589 |
| Control x SW+M |  | 7.837 | **0.049** |
| SW x SW+M |  | 0.520 | 0.511 |
| Ambient SW x Control |  | 140.398 | **2.641x10^-4^** |
| Ambient SW x SW |  | 18.951 | **0.012** |
| Ambient SW x SW+M |  | 173.935 | **1.910x10^-4^** |
| Control x SW | Order Rhodospirillales | 1.457 | 0.294 |
| Control x SW+M |  | 0.020 | 0.895 |
| SW x SW+M |  | 1.382 | 0.305 |
| Ambient SW x Control |  | 128.811 | **3.436x10^-4^** |
| Ambient SW x SW |  | 63.723 | **0.001** |
| Ambient SW x SW+M |  | 213.023 | **1.282x10^-4^** |
| Control x SW | Order Puniceispirillales | 95.522 | **6.141x10^-4^** |
| Control x SW+M |  | 2.099 | 0.221 |
| SW x SW+M |  | 51.310 | **0.002** |
| Ambient SW x Control |  | 138.832 | **2.969x10^-4^** |
| Ambient SW x SW |  | 6.115 | 0.069 |
| Ambient SW x SW+M |  | 64.959 | **0.001** |
| Control x SW | Order Rhizobiales | 49.144 | **0.002** |
| Control x SW+M |  | 0.010 | 0.927 |
| SW x SW+M |  | 40.902 | **0.003** |
| Ambient SW x Control |  | 116.132 | **4.205x10^-4^** |
| Ambient SW x SW |  | 301.024 | **6.478x10^-5^** |
| Ambient SW x SW+M |  | 82.659 | **8.116x10^-4^** |
| Control x SW | Order Vibrionales | 2.441 | 0.193 |
| Control x SW+M |  | 11.631 | **0.027** |
| SW x SW+M |  | 4.991 | 0.089 |
| Ambient SW x Control |  | 3.748 | 0.125 |
| Ambient SW x SW |  | 2.245 | 0.208 |
| Ambient SW x SW+M |  | 11.393 | **0.028** |
| Control x SW | Genus *Catenococcus* | 3.067 | 0.155 |
| Control x SW+M |  | 11.000 | **0.029** |
| SW x SW+M |  | 6.489 | 0.063 |
| Ambient SW x Control |  | 1.305 | 0.317 |
| Ambient SW x SW |  | 2.948 | 0.161 |
| Ambient SW x SW+M |  | 10.920 | **0.030** |

**Table S3** Sample type, code, and individual alpha-diversity (Shannon’s H’ diversity, richness, Pielou’s evenness) metrics. No hold time in seawater or menthol (Control); 3 hours in seawater only (SW); 3 hours in seawater and menthol (SW+M); ambient seawater filters (Ambient SW).

| Sample Type | Code | Diversity | Richness | Evenness |
| --- | --- | --- | --- | --- |
| Control | 8Jul23-2-1A | 2.337 | 1650 | 0.315 |
|  | 8Jul23-2-1B | 2.992 | 2071 | 0.392 |
|  | 8Jul23-2-1C | 2.274 | 1525 | 0.310 |
| SW | 8Jul23-2-1D | 2.287 | 1070 | 0.328 |
|  | 8Jul23-2-1E | 2.195 | 559 | 0.347 |
|  | 8Jul23-2-1F | 2.186 | 793 | 0.327 |
| SW+M | 8Jul23-2-1G | 2.306 | 717 | 0.351 |
|  | 8Jul23-2-1H | 2.561 | 1354 | 0.355 |
|  | 8Jul23-2-1I | 2.733 | 1468 | 0.375 |
| Ambient SW | 8Jul23-2-1AW | 3.960 | 1491 | 0.542 |
|  | 8Jul23-2-1BW | 3.780 | 1368 | 0.524 |
|  | 8Jul23-2-1CW | 3.783 | 1445 | 0.520 |

**Table S4** The number (# of OTUs) and percentage (%, out of 6,612 total OTUs in study) of significantly differential OTUs between sample types based on multiple linear regression with covariate adjustment performed in MicrobiomeAnalyst.

| Pairwise Comparison | # of OTUs | % |
| --- | --- | --- |
| Control x SW+M | 65 | 0.98 |
| Control x SW | 122 | 1.85 |
| SW x SW+M | 54 | 0.82 |
| Ambient SW x Control | 795 | 12.02 |
| Ambient SW x SW | 618 | 9.35 |
| Ambient SW x SW+M | 575 | 8.70 |

**Table S5** Top ten most abundant OTUs that were significantly differential for pairwise comparisons using multiple linear regression with covariate adjustment between Control, SW, and SW+M treatments. P-values (p) and FDR-adjusted p-values (FDR) are reported for each OTU (#), as well as the abundance per sample collected on July 8, 2023. Shaded cells indicate which groups were being compared in the pairwise analysis.

| Pairwise Comparison | OTU # | p | FDR | Control | | | | SW | | | SW+M | | | Ambient SW | | |
| --- | --- | --- | --- | --- | --- | --- | --- | --- | --- | --- | --- | --- | --- | --- | --- | --- |
| Control x SW+M |  |  |  | 2-1A | 2-1B | | 2-1C | 2-1D | 2-1E | 2-1F | 2-1G | 2-1H | 2-1I | AW | BW | CW |
|  | 10 | 1.56x10^-5^ | 1.27x10^-3^ | 167 | 21 | | 34 | 1275 | 5841 | 1185 | 9840 | 23292 | 10562 | 136 | 130 | 117 |
|  | 53 | 3.03x10^-4^ | 0.01 | 5 | 9 | | 3 | 32 | 18 | 35 | 33 | 29 | 24 | 717 | 539 | 596 |
|  | 70 | 4.92x10^-7^ | 6.88x10^-5^ | 0 | 0 | | 0 | 2 | 2 | 0 | 524 | 1013 | 230 | 1 | 0 | 0 |
|  | 77 | 2.81x10^-4^ | 0.01 | 0 | 0 | | 0 | 1 | 2 | 0 | 3 | 3 | 6 | 415 | 364 | 330 |
|  | 115 | 7.64x10^-4^ | 0.03 | 3 | 8 | | 0 | 9 | 5 | 0 | 281 | 137 | 406 | 0 | 0 | 1 |
|  | 150 | 4.18x10^-6^ | 4.23x10^-4^ | 0 | 0 | | 0 | 1 | 6 | 1 | 178 | 112 | 167 | 2 | 4 | 1 |
|  | 285 | 1.71x10^-3^ | 4.90x10^-2^ | 14 | 133 | | 4 | 0 | 0 | 0 | 0 | 0 | 1 | 0 | 0 | 0 |
|  | 328 | 4.14x10^-4^ | 0.02 | 1 | 0 | | 0 | 12 | 2 | 0 | 21 | 53 | 41 | 0 | 0 | 0 |
|  | 387 | 4.88x10^-5^ | 3.12x10^-3^ | 0 | 0 | | 1 | 0 | 0 | 0 | 20 | 10 | 83 | 0 | 0 | 0 |
|  | 639 | 4.88x10^-5^ | 3.12x10^-3^ | 9 | 16 | | 9 | 0 | 0 | 0 | 3 | 4 | 3 | 0 | 0 | 0 |
| Control x SW |  |  |  |  |  | |  |  |  |  |  |  |  |  |  |  |
|  | 4 | 1.42x10^-5^ | 1.08x10^-3^ | 15169 | 16889 | | 14588 | 7469 | 8649 | 8158 | 20339 | 15791 | 17180 | 360 | 350 | 333 |
|  | 6 | 5.38x10^-5^ | 3.10x10^-3^ | 6411 | 8480 | | 7409 | 14825 | 18200 | 17354 | 8638 | 6038 | 7978 | 0 | 0 | 0 |
|  | 10 | 2.77x10^-4^ | 0.01 | 167 | 21 | | 34 | 1275 | 5841 | 1185 | 9840 | 23292 | 10562 | 136 | 130 | 117 |
|  | 13 | 1.11x10^-4^ | 5.21x10^-3^ | 1010 | 1461 | | 1571 | 2976 | 3681 | 3813 | 1102 | 1636 | 1338 | 0 | 0 | 0 |
|  | 37 | 1.48x10^-4^ | 6.65x10^-3^ | 0 | 7 | | 15 | 512 | 1518 | 302 | 7 | 68 | 14 | 611 | 382 | 415 |
|  | 53 | 3.59x10^-4^ | 0.01 | 5 | 9 | | 3 | 32 | 18 | 35 | 33 | 29 | 24 | 717 | 539 | 596 |
|  | 102 | 1.29x10^-3^ | 0.03 | 7 | 2 | | 2 | 86 | 84 | 34 | 4 | 1 | 11 | 194 | 201 | 115 |
|  | 121 | 7.75x10^-6^ | 6.55x10^-4^ | 63 | 272 | | 64 | 0 | 1 | 1 | 94 | 51 | 215 | 0 | 0 | 0 |
|  | 125 | 1.60x10^-5^ | 1.19x10^-3^ | 153 | 100 | | 104 | 0 | 0 | 0 | 83 | 101 | 110 | 0 | 0 | 0 |
|  | 133 | 5.38x10^-5^ | 3.10x10^-3^ | 81 | 89 | | 121 | 0 | 0 | 0 | 108 | 74 | 106 | 0 | 0 | 0 |
| SW x SW+M |  |  |  |  |  | |  |  |  |  |  |  |  |  |  |  |
|  | 4 | 3.65x10^-6^ | 3.81x10^-4^ | 15169 | 16889 | 14588 | | 7469 | 8649 | 8158 | 20339 | 15791 | 17180 | 360 | 350 | 333 |
|  | 6 | 5.92x10^-5^ | 3.63x10^-3^ | 6411 | 8480 | 7409 | | 14825 | 18200 | 17354 | 8638 | 6038 | 7978 | 0 | 0 | 0 |
|  | 13 | 1.22x10^-4^ | 6.32x10^-3^ | 1010 | 1461 | 1571 | | 2976 | 3681 | 3813 | 1102 | 1636 | 1338 | 0 | 0 | 0 |
|  | 37 | 7.53x10^-4^ | 0.03 | 0 | 7 | 15 | | 512 | 1518 | 302 | 7 | 68 | 14 | 611 | 382 | 415 |
|  | 70 | 1.49x10^-6^ | 1.73x10^-4^ | 0 | 0 | 0 | | 2 | 2 | 0 | 524 | 1013 | 230 | 1 | 0 | 0 |
|  | 94 | 1.48x10^-4^ | 7.38x10^-3^ | 140 | 133 | | 120 | 0 | 0 | 0 | 227 | 126 | 297 | 0 | 0 | 0 |
|  | 115 | 1.02x10^-3^ | 0.03 | 3 | 8 | | 0 | 9 | 5 | 0 | 281 | 137 | 406 | 0 | 0 | 1 |
|  | 121 | 8.00x10^-6^ | 7.02x10^-4^ | 63 | 272 | | 64 | 0 | 1 | 1 | 94 | 51 | 215 | 0 | 0 | 0 |
|  | 125 | 6.49x10^-5^ | 3.91x10^-3^ | 153 | 100 | | 104 | 0 | 0 | 0 | 83 | 101 | 110 | 0 | 0 | 0 |
|  | 133 | 5.80x10^-5^ | 3.57x10^-3^ | 81 | 89 | | 121 | 0 | 0 | 0 | 108 | 74 | 106 | 0 | 0 | 0 |
